# Research on the performance of different waterproof bonding layer materials for steel bridge decks

**DOI:** 10.1038/s41598-025-23512-y

**Published:** 2025-11-13

**Authors:** Huang Biao, Shi Jie, Chen Songqiang

**Affiliations:** 1 Wenzhou Lucheng District Urban Construction Center, Wen Zhou, 325000 Zhejiang China; 2 College of Construction Engineering, Zhejiang University, Hangzhou, 310015 Zhejiang China; 3 Department of Civil Engineering, School of Engineering, Hangzhou City University, Hangzhou, 310015 Zhejiang China

**Keywords:** Steel bridge deck, Waterproof bonding layer, Surface energy theory, Bonding strength, Interlayer shear test, Engineering, Materials science

## Abstract

This study investigates the performance of three commonly used waterproof bonding layer materials for steel bridge decks—solvent-based rubber asphalt, methyl methacrylate (MMA) resin, and epoxy resin—through surface energy theory, pull-off tests, and interlayer shear tests. The results indicate that MMA resin exhibits the highest surface energy parameters, followed by epoxy resin, while solvent-based rubber asphalt shows the lowest values. Pull-off tests reveal that epoxy resin achieves peak bond strength (10.22 MPa) after 60 h of curing at 25 °C, whereas solvent-based rubber asphalt performs best at an application rate of 0.4 kg/m^2^. Interlayer shear tests demonstrate that epoxy resin provides the highest shear strength, which increases with loading rate but decreases significantly at elevated temperatures. Additionally, temperature significantly affects bonding performance, with epoxy and MMA resins outperforming solvent-based rubber asphalt under high-temperature conditions. This research provides a theoretical basis for material selection and construction parameter optimization of waterproof bonding layers for steel bridge decks.

## Introduction

Steel bridges are widely used due to their advantages such as light self-weight, strong spanning capacity, and convenient construction, and have accumulated substantial theoretical and practical experience. The orthotropic steel deck structure, commonly employed in steel box girders, is formed by welding mutually perpendicular components including longitudinal stiffeners, vertical webs, transverse diaphragms, and deck plates^1,2^. The orthotropic steel deck directly participates in load-bearing, effectively reducing the self-weight of the deck and improving the bridge’s mechanical performance. However, as a large thin-walled spatial structure with complex construction, it is prone to fatigue cracking under vehicle wheel loads. Negative bending moments develop above the diaphragms and longitudinal stiffeners, while the steel box girder undergoes expansion–contraction bending, torsion, and other deformations. This induces non-uniform stress and strain within the pavement layer, leading to fatigue cracking^3,4^. Previous research indicates that heavy vehicle loads can significantly increase shear stress levels in the bridge deck pavement, particularly in summer when high temperatures exacerbate structural damage. Among various factors, interlayer bonding performance is a critical determinant of this behavior^5,6^.

As one of the key bridge pavement materials, the waterproof bonding layer serves as both a protective coating and an adhesive between the pavement layer and the steel plate, preventing water penetration^7^. It not only bonds the bridge deck and asphalt pavement but also coordinates deformation and protects the steel plate from corrosion, playing a vital role in ensuring the quality and durability of the deck pavement[^8,9^]. The failure of waterproof bonding significantly impacts the service life of steel bridges^10,11^. Extensive practice has demonstrated that the loss of bonding strength at the interface between the asphalt mixture and the steel plate is one of the primary causes of steel bridge deck damage^12^.

Surface energy theory provides a crucial scientific and quantitative basis for selecting waterproof bonding materials for bridge decks^13,14^. In practice, by measuring the surface energy parameters of asphalt, waterproofing membrane materials, and the concrete deck, the adhesive strength and the energy of debonding in the presence of water can be theoretically calculated and compared^15,16^. This process elevates material selection from empirical judgment to a quantifiable scientific decision-making. Its core practical significance lies in the ability to proactively predict the long-term in-service performance of material combinations under traffic loading and environmental erosion. By optimizing the selection of materials with high surface energy compatibility, the interlayer shear strength and resistance to moisture-induced damage can be significantly enhanced, thereby directly guiding the choice of the most durable bonding system to ensure the longevity and high reliability of the bridge deck structure^17,18^.

Common waterproof bonding materials for steel bridge decks include: Methyl methacrylate resin^19^, Epoxy resin^20^, Solvent-based adhesives, Modified asphalt^21^. Solvent-based adhesives are formed by dissolving asphalt and rubber polymers and coating them onto the steel plate interface. Based on the principles of similar compatibility and complementary properties, they create a tough and dense network structure. The waterproof membrane partially melts and blends with the asphalt concrete, achieving effective bonding between the deck and asphalt pavement^22^. These adhesives bond to the steel plate through physical processes, offering a degree of reversibility and enabling stronger cohesion between upper and lower pavement layers. V. Bagiatis^23^ studied the effect of atmospheric pressure plasma treatment (APPT) on the performance enhancement of poly (methyl methacrylate) (PMMA) substrates. Li et al.^24^ conducted shear tests and found that the shear strength of epoxy resin waterproof bonding layers is influenced by factors such as thickness, concrete surface roughness, aggregate size, and test temperature. Feng et al.^25^ investigated the effect of steel deck roughness on bonding strength using pull-off tests, revealing that epoxy waterproof coatings exhibit the strongest bonding when the roughness is 60 μm. Wang^26^ simulated the mechanical performance of rubber asphalt waterproof layers under vehicle loads using pull-off and direct shear tests. Their results showed that rubber asphalt performs well as a waterproof adhesive, but its performance degrades with increasing temperature. Although the quality of waterproof adhesive systems has improved over the years, most research has focused on optimizing specific aspects such as shear strength or fatigue resistance. Evaluating the overall performance of adhesive systems requires considerable effort, and further optimization is needed based on temperature and cost analysis.

Therefore, this study selects three commonly used waterproof bonding materials for steel bridge decks. The adhesion work between these materials and steel plates is analyzed using surface energy theory. Pull-off tests are conducted to evaluate the bonding strength between the materials and steel plates, and interlayer shear tests are performed to assess the bonding strength between steel plates and asphalt mixtures under different waterproof bonding materials. This comprehensive analysis aims to elucidate the influence of waterproof bonding materials on the bonding strength of steel bridge decks.

## Raw materials and testing methods

### Raw materials

Three commonly used waterproof bonding layer materials were selected for analysis: solvent-based asphalt binder, methyl methacrylate, and epoxy resin. The main technical specifications of these materials are presented in Tables 1, 2, 3, and 4. All technical specifications meet or exceed the required standards. The solvent-based rubber binder demonstrates particularly high elongation at break, while the methyl methacrylate shows excellent drying performance. The epoxy resin system exhibits optimal viscosity and thermal stability characteristics.

**Table 1:** Solvent-based rubber waterproof bonding layer.

Technical indicator	Unit	Requirement	Measured value
Solid content	%	≥ 50	72
Surface drying time (23°C)	h	≤ 1.5	1.2
Complete drying time (23°C)	h	≤ 8	7.2
Water impermeability (0.3MPa, 30min)	–	Impermeable	Impermeable
Tensile strength	MPa	≥ 0.4	0.46
Elongation at break	%	≥ 900	932

**Table 2:** Methyl methacrylate waterproof bonding layer.

Technical Indicator	Unit	Requirement	Measured value
Solid content	%	≥ 95	107
Surface drying time (23 °C)	h	≤ 0.5	0.3
Complete drying time (23 °C)	h	≤ 1	0.5
Water impermeability (0.3 MPa, 30 min)	–	Impermeable	Impermeable
Low-temperature flexibility	–	No cracking	No cracking

**Table 3:** Component A of epoxy resin waterproof bonding layer.

Technical indicator	Unit	Requirement	Measured value
Viscosity	10^–3^ Pa s	6000 ~ 25,000	10,500
Epoxy equivalent weight	g	185 ~ 210	189
Color	Gardner	≤ 4	2
Water content	%	≤ 0.05	0.03
Flash point	°C	≥ 200	275

**Table 4:** Component B of epoxy resin waterproof bonding layer.

Technical indicator	Unit	Requirement	Measured value
Viscosity (25°C)	10^–3^ Pa s	≥ 800	850
Acid value	mg KOH/g	≤ 200	159
Water content	%	≤ 0.05	0.03
Flash point	°C	≥ 250	275
Specific gravity (25°C)	g/cm^3^	1.00 ± 0.15	1.09

### Test method

1. Contact Angle Test.

**Fig. 1:**
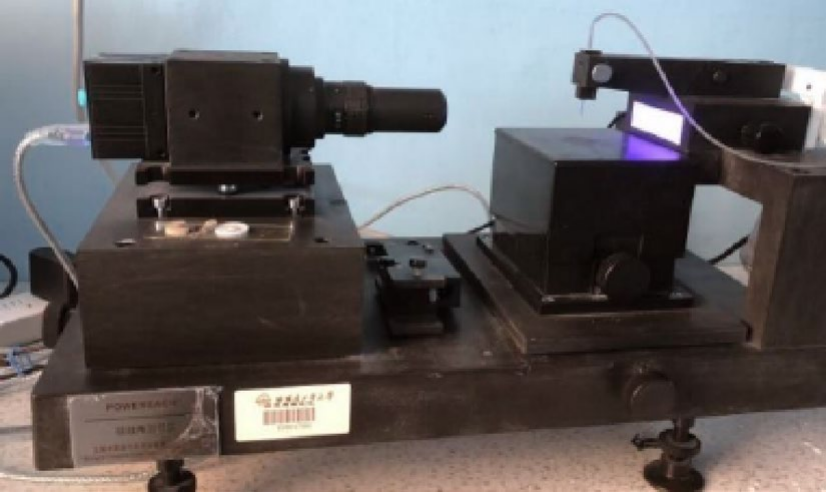
Contact angle goniometer.

**Fig. 2:**
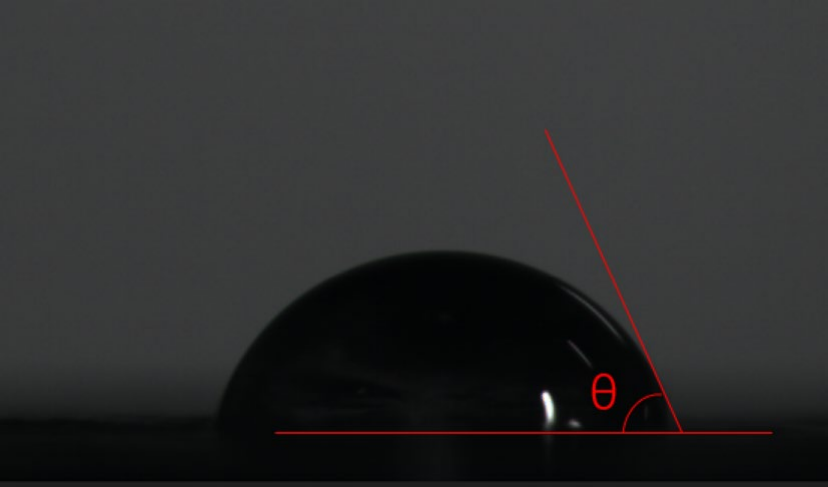
Contact angle.

The Contact Angle Goniometer is used to measure the contact angle of liquids on solid surfaces to evaluate material properties such as wettability, surface energy, and cleanliness. Standard material specimens are prepared and placed on the test platform. A liquid with known surface energy parameters is dispensed onto the surface, and side-view images are captured using a high-speed camera. The contact angle is calculated using either the Young–Laplace equation or the Tangent Method, as illustrated in the Figs. 1 and 2.

(2) Pull-Off Test.

**Fig. 3:**
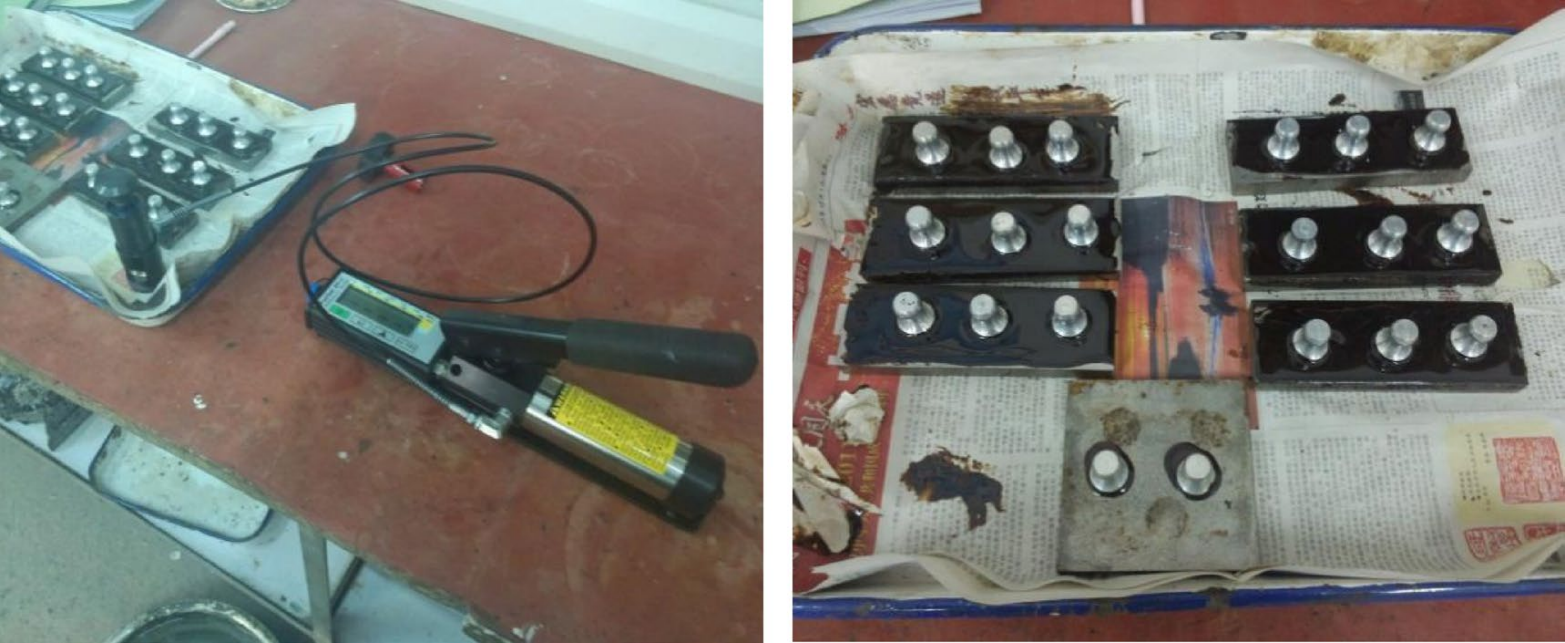
Pull-off testing machine and specimen.

The pull-off test is a critical quality control indicator for evaluating the bonding strength between the waterproof bonding layer and the steel bridge deck. The steel plate is first sandblasted, coated with the waterproof bonding material, and then fitted with a pull-off stub. The bonding strength is measured using a pull-off tester, as shown in Fig. 3.

(3) Interlayer Shear Test.

**Fig. 4:**
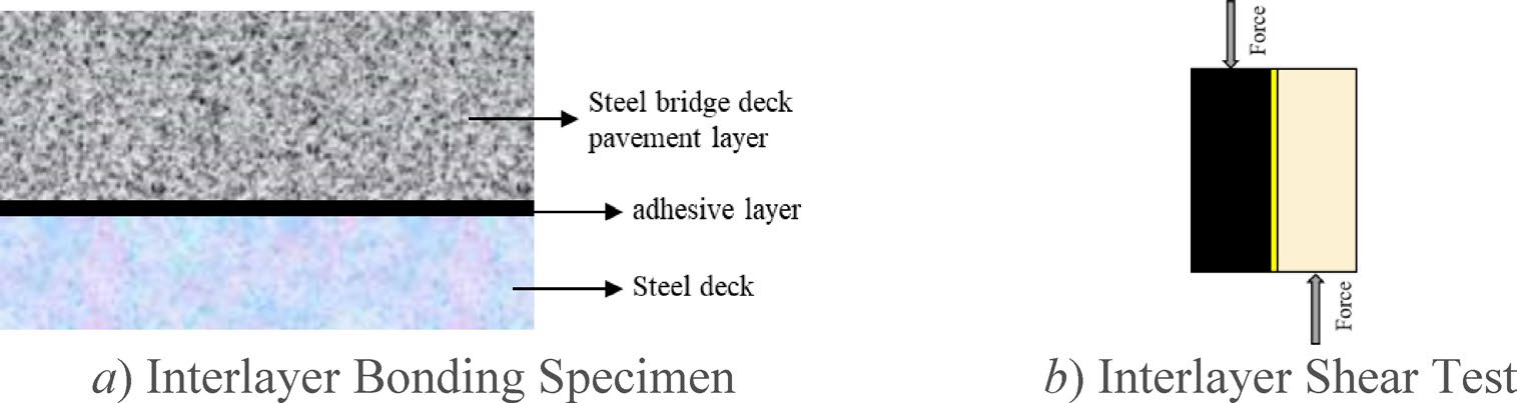
Interlayer shear test.

Following the requirements of Technical Specifications for Design and Construction of Highway Steel Bridge Deck Pavements (JTG/T 3364-02-2019), the steel plates are cleaned, and shear test specimens are prepared using the optimal coating amounts recommended in the previous section for three types of waterproof bonding materials. The MTS universal testing machine is employed to measure the shear strength between the asphalt mixture and the steel plate. The specimen preparation process is illustrated in Fig. 4.

## Research on the bonding mechanism of waterproof adhesive layer materials based on surface energy theory

### Surface energy theory

The heterogeneous system encompasses the results of interactions among three phases, forming five types of interfaces: gas–liquid, gas–solid, liquid–liquid, liquid–solid, and solid–solid. Generally, the interfaces where the solid or liquid phase contacts the gas phase are referred to as surfaces. Relatively speaking, the influence of the gas phase on heterogeneous systems is minor, especially in road engineering. Therefore, the primary focus is on the interfaces formed between the solid and liquid phases. These interfacial regions significantly affect the system’s behavior, making their study particularly important.

The surface free energy is composed of a dispersion component ($\gamma^{LW}$) and a polar component ($\gamma^{AB}$). The dispersion component, also known as van der Waals forces, consists of Keesom orientation forces, Debye induction forces, and London dispersion forces. The polar component, also referred to as Lewis acid–base interactions, is formed by Lewis acid and Lewis base forces[^27,28^]. Their relationship is expressed by the following Eq. (1).

1 $$\gamma = \gamma^{LW} + \gamma^{AB} = \gamma^{LW} + 2\sqrt {\gamma^{ + } \gamma^{ - } }$$

According to the literature[^29^], the interfacial free energy between the steel bridge deck and the waterproof bonding layer can be expressed by Eq. (2).

2 $$\gamma_{sw} = \gamma_{s} + \gamma_{w} - 2\sqrt {\gamma_{s}^{LW} \gamma_{w}^{LW} } - 2\sqrt {\gamma_{s}^{AB} \gamma_{w}^{AB} }$$

where $\gamma_{s}^{LW}$ and $\gamma_{w}^{LW}$ represent the dispersive components of the steel bridge deck and the waterproof bonding layer, respectively, and $\gamma_{s}^{AB}$ and $\gamma_{w}^{AB}$ denote the polar components of the waterproof bonding layer and the steel bridge deck, respectively.

The adhesion model for the steel bridge deck/waterproof bonding layer system is as follows:

3 $$W_{sw} = \gamma_{s} (1 + \cos \theta ) = 2\sqrt {\gamma_{s}^{LW} \gamma_{w}^{LW} } + 2\sqrt {\gamma_{s}^{ + } \gamma_{w}^{ - } } + 2\sqrt {\gamma_{s}^{ - } \gamma_{w}^{ + } }$$

It should be noted that Eq. (3) serves two purposes in this study:

1. By using three test liquids with known surface energy parameters as the liquid phase and combining them with contact angle data measured via the sessile drop method, substituting these into Eq. (3) allows solving the system of equations to determine the unknown surface energy parameters of the solid phase (i.e., solid asphalt and aggregates).
2. By incorporating the known surface energy parameters of the steel bridge deck and waterproof bonding layer materials into Eq. (3), the adhesion work of the asphalt-aggregate system can be directly obtained.

### Surface energy parameter testing

By measuring the contact angles between three solutions with known surface energy parameters (as shown in Table 5) and the steel bridge deck/waterproof bonding layer materials, the surface energy parameters of the materials can be calculated by solving the equations. Subsequently, the adhesion work between the bridge deck steel plate and the waterproof bonding layer material can be determined using Eq. (3), which serves as an indicator for evaluating the adhesive performance of the waterproof bonding layer. The steel plate used was identical to the Q345 steel plate employed in bridges, with a diameter of 100 mm, a height of 10 mm, and surface treatment including sandblasting (cleanliness grade Sa2.5) and a roughness of 80 ~ 120 μm. The surface was evenly divided into six sectors using white paint, and 12 valid measurements were taken per specimen, with the average value recorded.

**Table 5:** Surface energy parameters of common test liquids.

Liquid name	$\gamma_{L}$(mJ/m^2^)	$\gamma_{L}^{LW}$(mJ/m^2^)	$\gamma_{L}^{AB}$(mJ/m^2^)	$\gamma_{L}^{ + }$(mJ/m^2^)	$\overline{{\gamma_{L}^{ - } }}$(mJ/m^2^)
Deionized Water	72.8	21.8	51	25.5	25.5
Glycerol	64	34	30	3.92	57.4
Formamide	58	39	19	2.28	39.6

The experiments were conducted at room temperature, and the test sample was placed on the workstation, and a droplet of the test liquid (with known surface energy parameters) was dispensed using a syringe needle. The workstation was finely adjusted to allow gentle contact between the droplet and the sample surface, forming a sessile drop. After stabilization, the droplet-material interface was captured via imaging, and the contact angle was measured using a JC2000D contact angle goniometer. The contact angles between different liquids and the waterproof bonding layer are presented in Table 6.

**Table 6:** Contact angles between waterproof bonding layers and test liquids $\theta (^{{\text{o}}} )$.

Waterproof bonding layer type	Deionized water	Glycerol	Formamide
Epoxy resin	88.2	79.5	63.2
Methacrylic resin	89.8	80.8	64.6
Solvent-based rubber	81.3	70.0	59.7

**Fig. 5:**
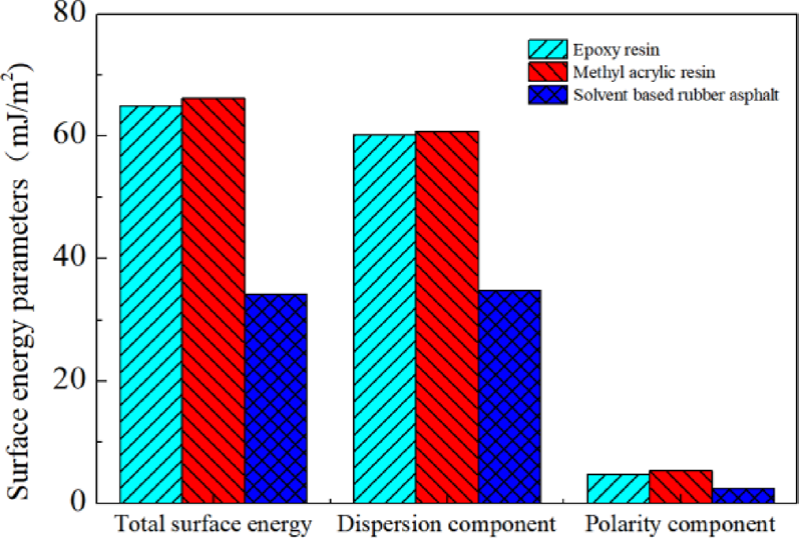
Surface energy parameters of different waterproof bonding layer materials.

In the theory of Eq. (3), the total surface energy (γ) is divided into three components: the van der Waals component (γ^*LW*^), the acidic component (γ^+^), and the basic component (γ^-^). These three components collectively constitute the adhesive interactions at the contact interface. Equation (3) serves as the fundamental equation for calculating the surface energy components of asphalt by measuring contact angles. In this equation, the waterproof bonding layer to be tested represents the solid phase (*S*), while the liquid phase (*L*) consists of probe liquids with known surface energy components. If the square roots of the three unknown surface energy components of the waterproof bonding layer are denoted as *x*_1_, *x*_2_, and *x*_3_, respectively, then Eq. (3) can be expressed as (Fig. 5):

4 $$\gamma_{L} (1 + \cos \theta ) = 2\sqrt {\gamma_{L}^{LW} } x_{1} + 2\sqrt {\gamma_{L}^{ + } } x_{2} + 2\sqrt {\gamma_{L}^{ - } } x_{3}$$

where *x*_1_ is $\sqrt {\gamma_{s}^{LW} }$, *x*_2_ is $\sqrt {\gamma_{s}^{ + } }$, and *x*_3_ is $\sqrt {\overline{\gamma }_{s} }$.

Based on Eq. (4) and the solution of the ternary linear equations using MATLAB, the obtained results are shown in Table 7 and Fig. 5. From the table, it can be observed that methacrylic resin has the highest surface energy parameters, followed by epoxy resin, while solvent-based rubber asphalt exhibits the lowest values. Using the same experimental method, the contact angles between Q345 steel plate and different test liquids were measured, as listed in Table 8.

**Table 7:** Surface energy parameters of different waterproof adhesive layer materials (mJ/m^2^).

Surface energy parameters	Epoxy resin	Methacrylic resin	Solvent based rubber asphalt
$\gamma_{{\text{L}}}$	64.98	66.19	34.25
$\gamma_{L}^{LW}$	60.23	60.82	34.84
$\gamma_{L}^{AB}$	4.75	5.37	2.41
$\gamma_{L}^{ + }$	2.01	2.45	0.27
$\overline{{\gamma_{L}^{ - } }}$	2.81	2.94	5.39

**Fig. 6:**
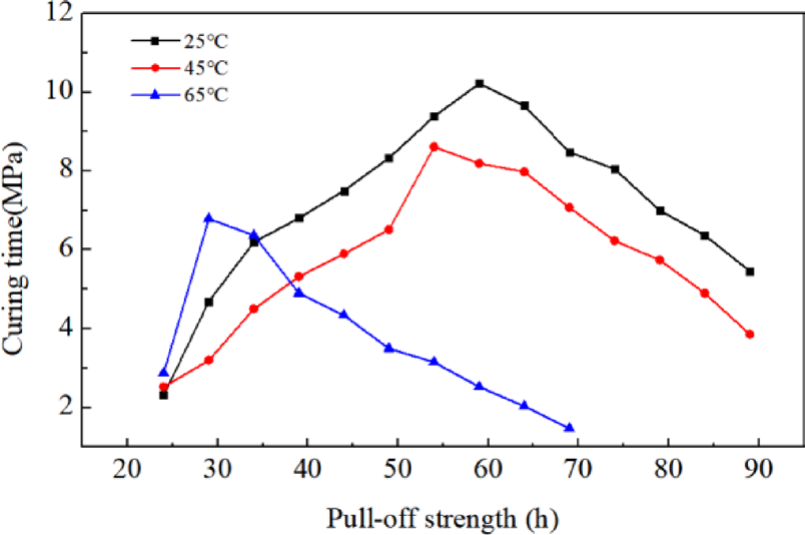
Tensile strength of epoxy resin and steel plate at different curing times.

**Table 8:** Contact angles $\theta (^{^\circ } )$ between Q345 steel and test liquids.

Material	Distilled Water	Glycerol	Formamide
Q345 Steel	68.5	59.2	43.0

By substituting the contact angles from Table 8 and the surface energy parameters of the test liquids into Eq. (4), the surface energy parameters of Q345 steel plate were calculated, as presented in Table 9.

**Table 9:** Surface energy parameters of Q345 steel(mJ/m^2^).

Material	$\gamma_{{\text{L}}}$	$\gamma_{L}^{LW}$	$\gamma_{L}^{AB}$	$\gamma_{L}^{ + }$	$\overline{{\gamma_{L}^{ - } }}$
Q345 Steel	56.56	55.48	1.08	0.03	9.77

### Adhesion work analysis

The adhesion work between different waterproof adhesive layer materials and the steel plate was calculated using Eq. (3), with the results presented in Table 10. From the table, it can be observed that methacrylic resin exhibits slightly higher adhesion work compared to epoxy resin. And solvent-based rubber asphalt demonstrates significantly lower adhesion work with the steel plate than both epoxy resin and methacrylic resin.

**Table 10:** Adhesion work between waterproof adhesive materials and steel plate.

Material	Methacrylic resin	Epoxy resin	Solvent based rubber asphalt
Adhesion work (mJ/m^2^)	125.1	126.6	92.0

## Tensile strength

### 1Epoxy resin

To eliminate the influence of non-test factors, the first step involves grinding the steel plate (20 × 20 cm) to ensure its roughness. The second step entails measuring out a certain amount of the main agent and curing agent, mixing them at a mass ratio of 1:1, and stirring thoroughly with a mixer. The epoxy adhesive is then evenly applied to the steel plate surface at a coating rate of 0.6 kg/m^2^ using a scraper. The steel plates are placed in ovens at 25°C, 45 °C, and 65 °C for a specified curing period, after which they are removed and left at room temperature. Pull-off studs, preheated in a 165°C oven, are then placed on the plates. After 1 h, a pull-off test is conducted to measure the adhesive strength. The specimens are subsequently returned to the oven for a second cycle, and the process is repeated accordingly.

The test results are shown in Table 11 and Fig. 6. From the data, it can be observed that as the curing time increases, the bond strength between the epoxy resin and the steel plate initially rises, peaks, and then gradually declines. Additionally, higher ambient temperatures lead to shorter curing times for the epoxy resin. At 25 °C, the epoxy resin approaches full curing at around 60 h, when it nears full curing at about 55 h at 45 °C. And it achieves near-full curing in approximately 30 h at 65°C. Elevated temperatures accelerate the cross-linking chemical reactions between epoxy resin components, thereby reducing curing time. Therefore, during the construction of epoxy resin waterproof bonding layers, it is essential to consider the ambient temperature and control the timing of the next structural layer application to ensure optimal bonding performance. Under normal temperatures, the construction interval should be kept within 60 h, with precautions against dust and contamination. In high-temperature conditions (e.g., summer), the interval should be shortened to 35 h or less.

**Table 11:** Tensile strength of epoxy resin at different temperatures and curing times (MPa).

Curing time /h	25 ℃	45 ℃	65 ℃
24	2.31	2.52	2.87
29	4.69	3.2	6.79
34	6.2	4.5	6.37
39	6.8	5.32	4.9
44	7.5	5.9	4.34
49	8.33	6.51	3.5
54	9.38	8.61	3.15
59	10.22	8.19	2.52
64	9.66	7.98	2.03
69	8.47	7.07	1.47
74	8.05	6.23	/
79	7	5.74	/
84	6.37	4.9	/
89	5.46	3.85	/

**Fig. 7:**
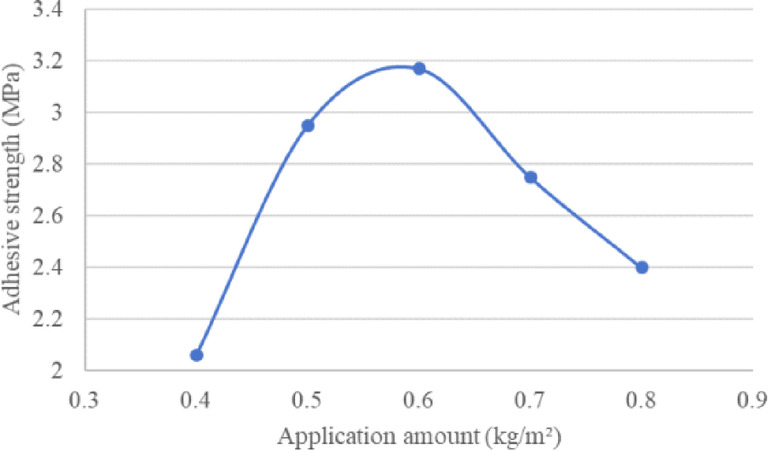
Adhesive strength of epoxy resin and steel plate with different application amounts.

To analyze the influence of epoxy resin application amount on bond strength, application amounts of 0.4 kg/m^2^, 0.5 kg/m^2^, 0.6 kg/m^2^, 0.7 kg/m^2^, and 0.8 kg/m^2^ were selected. The pull-off test results for the waterproof bonding layer material and steel plate are presented in Table 12 and Fig. 7. The data shows that within the tested range, bond strength initially increases with the application amount, peaking at around 0.6 kg/m^2^, after which further increases in application amount lead to a gradual decline in bond strength.

**Table 12:** Adhesive strength under different application amounts.

Application amount (kg/m^2^)	0.4	0.5	0.6	0.7	0.8
Adhesive strength (MPa)	2.06	2.95	3.17	2.75	2.4

### Solvent-based rubber asphalt

**Fig. 8:**
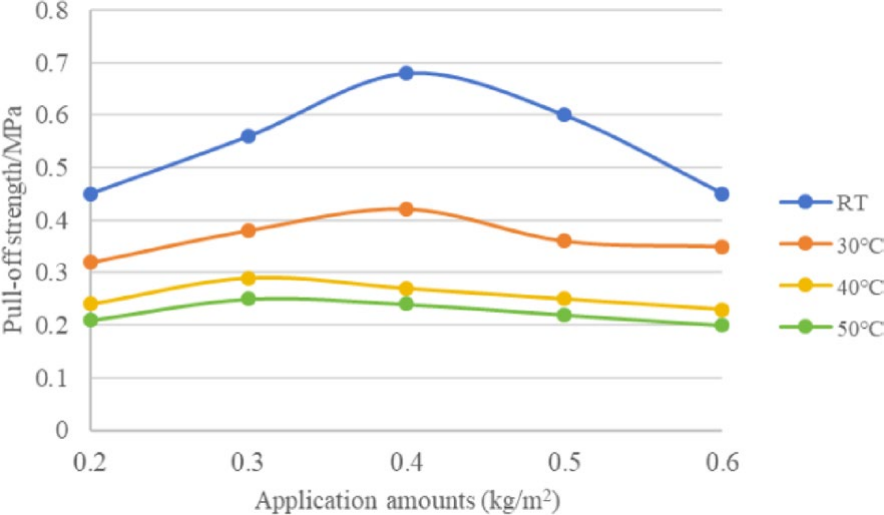
Pull-off strength between solvent-based rubber asphalt and steel plate with different application rates.

Unlike epoxy resin adhesives, the bonding process of solvent-based rubber asphalt does not involve chemical reactions. It simply requires waiting for the volatile components to evaporate. Therefore, for the pull-off test, the specimens were first left at room temperature for 60 h, then cured at a set temperature for 4 h before testing. The test results are shown in Table 13 and Fig.8. The data indicates that as the application amount increases, the pull-off strength between the solvent-based rubber asphalt and the steel plate first rises and then declines. At room temperature and 30 °C, the strength peaks at an application amount of around 0.4 kg/m^2^. At 40 °C and 50 °C, the peak occurs at approximately 0.3 kg/m^2^. Additionally, for the same application amount, the pull-off strength gradually decreases as the test temperature increases.

**Table 13:** Pull-off strength (MPa) between solvent-based rubber asphalt and steel plate under different temperatures and application amounts (Fig. 8).

Temperature (°C)	Application amounts (kg/m^2^)				
	0.2	0.3	0.4	0.5	0.6
Room temperature(RT)	0.45	0.56	0.68	0.6	0.45
30	0.32	0.38	0.42	0.36	0.35
40	0.24	0.29	0.27	0.25	0.23
50	0.21	0.25	0.24	0.22	0.2

### Methacrylic resin (MMA)

**Fig. 9:**
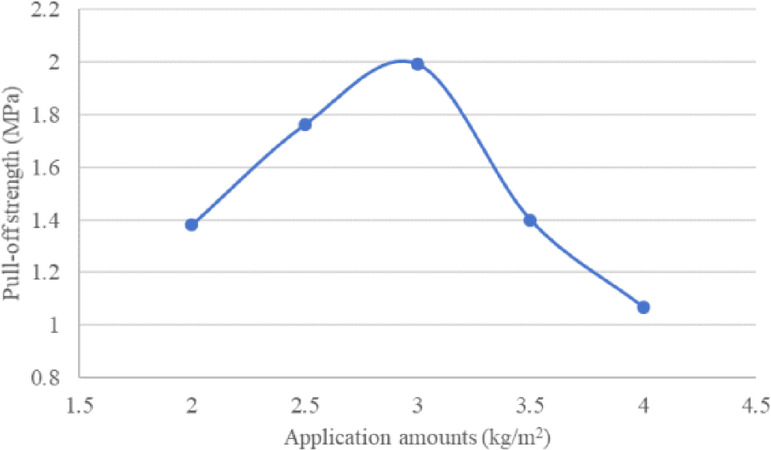
Pull-off strength of methacrylic resin to steel plate at different application rates.

The test results of MMA waterproof bonding layer materials with application rates of 2.0, 2.5, 3.0, 3.5, and 4.0 kg/m^2^ are presented in Table 14 and Fig. 9. The data demonstrate that the pull-off strength between the methacrylic resin and steel plate initially increases but then gradually decreases with higher application rates, reaching its maximum value at approximately 3.0 kg/m^2^.

**Table 14:** Pull-off strength of methacrylic resin to steel plate at different application rates (MPa).

Application amounts (kg/m^2^)	2	2.5	3	3.5	4
Pull-off strength (MPa)	1.38	1.76	1.99	1.4	1.07

## Interlaminar shear test

The primary distresses in bridge deck pavement layers—such as spalling, shoving, and upheaval—are predominantly caused by horizontal shear stresses induced under traffic loading. When these stresses exceed the shear resistance of the waterproof bonding layer, relative slippage occurs between the upper pavement layer and the underlying bridge deck, triggering distress formation. Consequently, the shear resistance of the waterproof bonding layer serves as a critical performance metric.

### Effect of loading rate on shear resistance

Based on the optimal application rates determined in the previous section, composite shear test specimens were prepared for three types of waterproof bonding materials. The shear resistance between these materials and steel plates was evaluated using an MTS universal testing machine. This study examined the influence of varying shear rates (1, 5, 10, 20, 30, and 50 mm/min) on the shear performance of the waterproof bonding materials. The test results are presented in Table 15 and Fig. 11.

**Table 15:** Shear strength between asphalt mixture and steel plate at different loading rates (MPa).

Loading rates (mm/min)		1	5	10	20	30	50
Shear strength	Epoxy resin	0.62	0.97	1.58	1.74	1.82	1.93
	Solvent based rubber asphalt	0.26	0.71	1.02	1.22	1.34	1.35
	Methacrylic resin	0.55	0.85	1.47	1.65	1.76	1.81

**Fig. 10:**
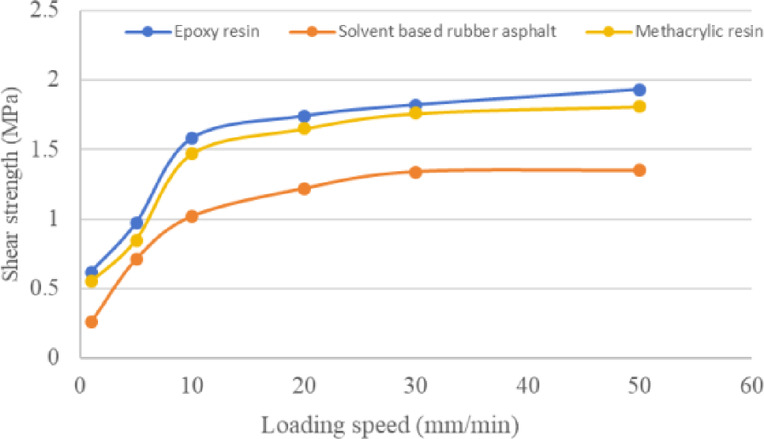
The influence of different loading rates on the shear strength between asphalt mixture and steel plate.

From Table 15 and Fig. 10, it can be seen that with the increase of loading speed, the interlayer bonding strength between asphalt mixture and steel plate under the three waterproof bonding layer materials gradually increases, and the increasing trend gradually decreases. The loading rate increased from 1 to 10 mm/min, and the shear strength growth rate between the asphalt mixture and the steel plate was relatively high. As the loading rate increased from 10 to 30 mm/min, the growth rate of shear strength gradually decreased. At a loading rate of 30 mm/min, the growth rate of shear strength was relatively slow. At the same loading rate, it can be seen that when epoxy resin is used as the waterproof bonding layer, the interlayer shear strength is the highest, followed by methyl acrylic resin, and solvent based rubber asphalt is the lowest.

### Effect of temperature on shear resistance

**Fig. 11:**
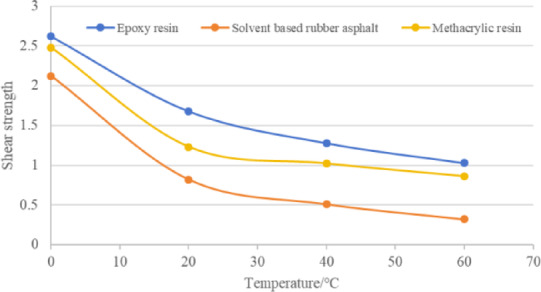
The effect of different test temperatures on the shear strength between asphalt mixture and steel plate.

Based on the recommended optimal application amount of waterproof bonding layer material in the previous section, shear composite specimens of three types of waterproof bonding layer materials were formed. The low temperature, normal temperature, and high temperature states were characterized at four temperatures of 0, 20, 40, and 60 ℃, respectively. The shear strength between asphalt mixture and steel plate was tested using MTS universal testing machine. The test results are shown in Table 16 and Fig. 11. It can be seen from this that as the temperature increases, the shear strength between the mixture and the steel plate gradually decreases, and the rate of decrease gradually slows down. At low temperatures, the interlayer shear strength of the three waterproof bonding materials is relatively close. As the temperature increases, the bond strength between epoxy resin and methyl acrylic resin is relatively close, while the difference in bond strength between solvent based rubber asphalt and the two materials becomes larger. At 0 ℃, the bond strength of epoxy resin is 20% greater than that of rubber asphalt, while at 60 ℃, the bond strength of epoxy resin is 220% greater than that of rubber asphalt.

**Table 16:** Shear strength between asphalt mixture and steel plate under different temperatures and waterproof bonding layer materials (MPa).

Temperatures /℃		0	20	40	60
Shear strength	Epoxy resin	2.62	1.68	1.28	1.03
	Solvent based rubber asphalt	2.12	0.82	0.51	0.32
	Methacrylic resin	2.48	1.23	1.02	0.86

## Analysis of three adhesion performance tests

Due to the fact that surface energy testing is conducted at room temperature, three types of waterproof bonding layer materials were selected to analyze the tensile strength and interlayer bonding strength of steel plates at room temperature, and the summarized data is shown in Table 17. From the table, it can be seen that the distribution pattern of macroscopic tensile strength and interlayer shear strength is consistent with the adhesion work, and there is a certain correlation between macro and micro adhesion properties. However, all three methods can evaluate the adhesion performance between different waterproof bonding layer materials and steel plates.

**Table 17:** Adhesion performance data between different materials and steel plates.

Material	Methacrylic resin	Epoxy resin	Solvent based rubber asphalt
Adhesion work (mJ/m^2^)	125.1	126.6	92.0
Tensile strength (MPa)	1.99	3.17	0.68
Interlaminar shear resistance (MPa)	1.81	1.93	1.35

## Conclusion

The paper analyzed the bonding performance of three waterproof bonding layer materials using surface energy theory, pull-out test, and interlayer shear test system. The conclusions are as follows:

1. The adhesion energy between epoxy resin and steel plate is 126.6 mJ/m^2^, the adhesion energy between acrylic resin and steel plate is 125.1 mJ/m^2^, and the adhesion energy between solvent based rubber asphalt and steel plate is 92.0 mJ/m^2^. The testing law of adhesion work is the same as the results of pull-out test and interlayer shear test, and can be applied to analyze the adhesion between waterproof bonding layer materials and steel plates.
2. Results indicate that the bond strength of epoxy resin is highly dependent on its curing conditions (time and temperature), peaking at 10.22 MPa after 60 h at 25 °C. In contrast, the optimal pull-off performance for solvent-based rubber asphalt and MMA resin is achieved at specific application rates of 0.4 kg/m^2^ and 3.0 kg/m^2^, respectively.
3. Epoxy resin demonstrates the highest interlayer shear strength (1.93 MPa at a loading rate of 50 mm/min), which exhibits a positive correlation with increased loading rates. Although elevated temperatures reduce the shear strength of all materials, both epoxy and MMA resins show superior high-temperature stability compared to solvent-based rubber asphalt.
4. The comprehensive evaluation suggests that epoxy resin is suitable for applications demanding the highest bond strength, albeit requiring strict control over construction temperature and curing time. Solvent-based rubber asphalt is more appropriate for ambient-temperature environments, with its performance reliant on an optimized application rate. MMA resin offers a balanced performance profile, but its effectiveness necessitates precise control over the application rate.

This study provides a scientific basis for selecting waterproof bonding materials and optimizing construction techniques for steel bridge deck pavements, thereby contributing to enhanced durability and service life of the paving system.

## Data Availability

Data is provided within the manuscript.

## References

[1] Guo, J., Zhang, G. & Cui, Y. Estimation of fatigue parameters and life prediction for orthotropic steel deck based on reverse markov theory [J]. *J. Struct. Eng.* **151**(5), 04025037 (2025).

[2] Cui, C. et al. Monitoring and detection of steel bridge diseases: A review [J]. *J. Traffic Trans. Eng. (English Edition)* **11**(2), 188 (2024).

[3] Thevendran, V. et al. Nonlinear analysis of steel conc-rete composite beams curved in plan [J]. *Finite Elem. Anal. Des.* **32**, 125–139 (1999).

[4] Chen, L., Qian, Z. & Wang, J. Multiscale numerical modeling of steel bridge deck pavements considering vehicle-pavement interaction [J]. *Int. J. Geomech.* **16**(1), 04015001 (2016).

[5] Liu, Y. et al. Interlayer residual stress analysis of steel bridge deck pavement during gussasphalt pavement paving [J]. *Construct. Build. Mater.* **324**, 126624–126636 (2022).

[6] Liu, Y., Qian, Z., Zheng, D. et al. Interlaminar thermal effect analysis of steel bridge deck pavement during gussasphalt mixture paving [J]. Int. J. Pavement Eng., 1–13 (2017).

[7] Zhang, M. et al. Research on the compatibility of waterproof layer materials and asphalt mixture for steel bridge deck[J]. *Construct. Build. Mater.* **269**, 121346 (2020).

[8] Chen, Q. et al. Modified waterborne epoxy as a cold pavement binder: Preparation and long-term working properties. *J. Mater. Civil Eng.* **33**(5), 04021079 (2021).

[9] Li, J. et al. Effect of the self-healing properties of asphalt mixture on the interlayer shear performance of bridge deck pavement [J]. *Constr. Build. Mater.* **16**, 378–392 (2023).

[10] Bouazaoui, L. et al. Experimental study of bonded steel concrete composite structures. *J. Construct. Steel Res.* **63**(9), 1268–1278 (2007).

[11] Souici, A. et al. Behaviour of both mechanically connected and bonded steel-concrete composite beams [J]. *Eng. Struct.* **49**, 11–23 (2013).

[12] Yifan, S. et al. Thermal and mechanical properties of natural fibrous nanoclay reinforced epoxy asphalt adhesives [J]. *Int. J. Adhesion Adhesives* **85**, 308–314 (2018).

[13] Xia, H. et al. Preparation and performance of durable waterproof adhesive layer for steel bridge deck based on self-stratification effect [J]. *Constr. Build. Mater.* **366**, 130133 (2023).

[14] Zhang, M. et al. Research on the compatibility of waterproof layer materials and asphalt mixture for steel bridge deck [J]. *Constr. Build. Mater.* **269**, 121346 (2021).

[15] Liu, X. et al. Experimental study on properties of epoxy binder and epoxy bonding chips layer for steel bridge deck pavement [J]. *Road Mater. Pavement Design* **23**(11), 2451–2465 (2022).

[16] Xia, H. et al. Preparation and properties of highly flexible acrylic-epoxy resin with self-stratification waterproof material for steel bridge decks [J]. *J. Mater. Civ. Eng.* **37**(9), 04025290 (2025).

[17] Qiu, Y. et al. Evaluation and optimization of bridge deck waterproof bonding system using multi-objective grey target decision method [J]. *Road Mater. Pavement Design* **21**(7), 1844–1858 (2020).

[18] Wang H, ** C, Liu H, et al. Rubber asphalt waterproof adhesive layer for steel bridge gussasphalt pavement [J]. Int. J. Struct. Integr. 12(2), 261–270. (2021).

[19] Zhang, M. Investigation on the effect of butyl acrylate (nBA) to improve the toughness properties of methacrylate-based waterproofing adhesive material (MMA) for the steel bridge deck [J]. *Adv. Mater. Sci. Eng.* **1**, 4310662 (2022).

[20] Xu, Y. et al. Shear fatigue performance of epoxy resin waterproof adhesive layer on steel bridge deck pavement [J]. *Front. Mater.* **7**, 618073 (2021).

[21] Zheng, Y. et al. Preparation and application of rubber modified emulsified asphalt. *Construct. Build. Mater.* **411**, 134540 (2024).

[22] Qiu, Y., An, S., Rahman, A. et al. Evaluation and optimization of bridge deck waterproof bonding system using multi-objective grey target decision method [J]. Road Mater. Pavement Design, 1–15 (2019).

[23] Bagiatis, V., Critchlow, G. W., Price, D. & Wang, S. The effect of atmospheric pressure plasma treatment (APPT) on the adhesive bonding of poly (methyl methacrylate)(PMMA)-to-glass using a polydimethylsiloxane (PDMS)-based adhesive. *Int. J. Adhesion Adhesives* **95**, 102405 (2019).

[24] Li, Y. et al. Research on failure mode and mechanism of different types of waterproof adhesive materials for bridge deck. *Int. J. Pavement Eng.* **16**(7), 602–608 (2015).

[25] Feng, D. C., Wei, W. D. & Zhan, S. T. Influence of moisture content of cement concrete on performance of waterproof adhesive layer in bridge deck [J]. *J. Highway Trans. Res. Develop. (Chinese Edition)* **30**(5), 47–51 (2013).

[26] Wang, J., Zhang, Z. & Li, Z. Performance evaluation of desulfurized rubber asphalt based on rheological and environmental effects [J]. *J. Mater. Civil Eng.* **32**(1), 04019330 (2020).

[27] Guo, P. et al. Adhesion of warm-mix recycled asphalt aggregate mixtures based on surface free energy theory [J]. *J. Mater. Civil Eng.* **31**(10), 04019209 (2019).

[28] Ding, Y. et al. Adhesion property of municipal solid waste incinerator bottom ash and limestone with asphalt based on surface energy theory [J]. *J. Adhesion* **100**(10), 25 (2024).

[29] Liu X, Zhou C, Cao Q, Chen L, Feng D (2022) Experimental study on properties of epoxy binder and epoxy bonding chips layer for steel bridge deck pavement. Road Materials and Pavement Design, 23(11), 2451-2465..

